# Toward phase contribution assessment in perioperative oncology: insights from NSCLC and a proposal for broader implementation

**DOI:** 10.1186/s40779-025-00622-2

**Published:** 2025-07-31

**Authors:** Hui-Yao Huang, Yan-Jie Han, Yu Tang, Ning Jiang, Da-Wei Wu, Ning Li

**Affiliations:** Department of Clinical Trials Center, National Cancer Center/National Clinical Research Center for Cancer/Cancer Hospital, Chinese Academy of Medical Sciences and Peking Union Medical College, Beijing, 100021 China

**Keywords:** Perioperative regimen, Phase contribution assessment, Clinical trial

Dear Editor,

On July 25, 2024, an Oncologic Drugs Advisory Committee meeting was held to discuss the design of the AEGEAN trial, which evaluated the perioperative use of durvalumab for resectable non-small cell lung cancer (NSCLC) [1]. The Food and Drug Administration (FDA) stated that the 2-arm trial was not designed to isolate contributions of neoadjuvant and adjuvant phases. Since the clinical benefit of adjuvant durvalumab remains uncertain, this limitation raises concerns about potential overtreatment. Therefore, it was proposed that future trial designs for perioperative regimens in resectable NSCLC should include within-trial assessments of phase-specific contributions, that is, an evaluation of the individual efficacy and toxicity of the neoadjuvant and adjuvant components within a combined perioperative regimen [2], although the FDA subsequently approved perioperative durvalumab based on the positive results of AEGEAN.

This position represents a significant advance in the regulatory approach to perioperative oncology trials, introducing new scrutiny regarding the phase-specific contributions of a single drug. This landmark event drives us to consider why phase-specific assessment was not previously required for single drugs. It may stem from the differences between chemotherapy and immunotherapy, and the cost concerns of pharmaceutical companies (detailed in the Additional file 1). Here, our discussion will primarily focus on two key questions: 1) Why has it become necessary to consider phase-specific contributions in perioperative treatment regimens for resectable NSCLC? 2) Should the principle of phase assessment be extended to perioperative protocols for other resectable tumors?

## Why is it important to mandate phase assessment in NSCLC perioperative regimens?

Currently, 5 immune checkpoint inhibitor (ICI)-based regimens have been approved for resectable NSCLC, including 2 adjuvant immunotherapies, 2 perioperative chemoimmunotherapies, and 1 neoadjuvant chemoimmunotherapy. However, the clinical benefit of adjuvant immunotherapy remains uncertain, with IMPower010 and Keynote-091 showing non-significant overall survival [hazard ratio (*HR*) = 0.97 and 0.87, respectively] [3, 4]. Similarly, the BR.31 trial with adjuvant durvalumab did not show an improvement in disease-free survival [5]. Cross-trial comparisons question the value of adjuvant phases in perioperative regimens as well, with neoadjuvant chemoimmunotherapy showing similar event-free survival to perioperative treatment in AEGEAN (*HR* = 0.66 vs. *HR* = 0.69) [1, 6]. Although the patient-level analysis of CheckMate 77 T and CheckMate 816 suggested a potential benefit from perioperative nivolumab, these findings are based on indirect comparisons that may introduce bias [7]. Therefore, the necessity of adjuvant immunotherapy is uncertain, highlighting the need for staged assessments.

In addition, given the expected long-term survival for resectable NSCLC patients, the potential late effects of immunotherapy need to be further evaluated. In the AEGEAN trial, 9% of patients receiving durvalumab plus chemotherapy experienced unresolved immune-related adverse events [1], which could negatively impact patients’ quality of life. Similarly, an indirect meta-analysis revealed significantly increased treatment-related adverse events when adding adjuvant immunotherapy to neoadjuvant therapy [8]. Moreover, the duration of 1-year for adjuvant therapy may be excessively long, imposing time and financial toxicity on patients. As shown in IMpower010 and KEYNOTE-091, only 65% and 52% of patients complete the 1-year adjuvant therapy, respectively, with toxicity and patient withdrawal as the most common reasons for treatment discontinuation [3, 4, 9]. Currently, improvements in cancer survival have led to increased attention to the issue of overtreatment and have placed greater emphasis on quality of life. In this context, additional adjuvant therapy may be detrimental in terms of toxicity and burden on life with a limited improvement in efficacy. While it has now been widely recognized that “less is more” in drug-dosing scenarios, we propose that this principle may also apply to drug duration, especially in potentially curative settings.

Financial toxicity is another factor to be considered. Rising medication costs have been a major concern globally, especially during the post-COVID-19 era. ICIs are among the most expensive medications. For patients, the high cost may be a barrier that limits access to potentially curative perioperative immunotherapy. Moreover, given the large population of patients with resectable NSCLC, prolonged adjuvant immunotherapy may impose a substantial financial burden on healthcare systems globally. Considering the payers’ perspective at the beginning of a trial could be beneficial, as they may incentivize trials that are potentially cost-saving or cost-effective [10]. Currently, the optimal duration of adjuvant immunotherapy and the patients who may benefit most from the adjuvant immunotherapy remain unclear. Therefore, it is important to identify subgroups of patients who are most likely to benefit from the full perioperative regimen. Such stratification could optimize resource allocation, reduce unnecessary treatment exposure, and improve the cost-effectiveness of care within healthcare systems.

## Whether phase assessment be mandated for perioperative protocols for other cancer types?

Currently, several perioperative regimens are under investigation for multiple tumor types. Although phase contribution assessment is currently limited to perioperative regimens for NSCLC, its potential extension to other resectable tumors is a matter of speculation. Regulatory ambiguity regarding this assessment may discourage sponsors from investing in research and development, potentially posing a barrier comparable to that of stringent regulatory demands. To mitigate this challenge, it is recommended that regulatory authorities provide timely and explicit guidance on the application of phase contribution assessment. Before clarification, sponsors are encouraged to engage in early dialogue with regulators before conducting perioperative trials.

Overall, we propose extending the phase contribution assessment to other cancer types. From a scientific and patient-centric perspective, the assessment of component contributions is an instinctive requirement to ensure that any component used is necessary. Especially, this standard does not seem to be a particularly high bar as it typically only requires showing that the combination effect exceeds any single component, and synergy is not required to be proven [2].

To address the regulatory requirements for phase contribution assessment while maintaining feasible drug development pathways, sequential and simultaneous development represent two alternative strategies. The sequential strategy involves validating the efficacy of adjuvant or neoadjuvant therapy individually, based on clinical needs. For instance, for regimens capable of achieving significant or complete pathological responses, neoadjuvant therapy alone might be sufficient and could avoid additional local therapy, as seen in rectal cancer with DNA mismatch repair deficiency. For early-stage tumors or those that cause minimal surgical injury, adjuvant therapy might be preferred. If perioperative therapy remains clinically necessary, further studies comparing perioperative approaches with neoadjuvant- or adjuvant-only regimens can be conducted. This strategy relies on a thorough understanding of tumor characteristics and drug mechanisms, ensuring evidence-based decisions for each stage. However, it is time-consuming and requires a large sample size, making it more suitable when confidence in perioperative regimens is limited and a gradual accumulation of evidence is needed. For the simultaneous strategy, the efficacy of both adjuvant and neoadjuvant therapies is evaluated concurrently. There are several alternative study designs proposed by the FDA, including a 4-arm design with perioperative, neoadjuvant, adjuvant, and standard of care (SOC) arms, as well as a 3-arm design featuring perioperative, neoadjuvant, and SOC arms [2]. Although a 4-arm factorial design is the optimal design to evaluate each phase of a perioperative approach, a 3-arm trial design is deemed more practical. Compared with the sequential strategy, the time and total sample size required for the simultaneous strategy using a 3-arm trial design could be spared, especially when a statistical difference between the perioperative and the neoadjuvant arms may not be required [2]. More importantly, in the 3-arm design, approval could be expected if either experimental arm shows a better risk–benefit profile than SOC. Other innovative trial design options may also be considered, including adaptive trial designs or re-randomization, although they can be more challenging operationally.

Finally, regulatory requirements should be informed by patient-centered principles, aiming to support the development of safer, more effective, and cost-effective treatment options, rather than remaining rigid. The decision concerning risk–benefit is not as easy for a newly diagnosed patient with curative cancer and can vary by cancer type. After all, immunotherapies have been approved for adjuvant and/or neoadjuvant therapy in only 5 cancer types: NSCLC, urothelial carcinoma, melanoma, esophageal cancer or gastroesophageal junction cancer, and triple-negative breast cancer. To ensure that the related trial design of the future is optimized, we should also seek to guarantee that patient and healthcare professional perspectives are routinely integrated. Engaging key stakeholders (including patients and health care professionals) early in the trial development process to codesign key trial features is thus likely to improve trial design and conduct, ultimately leading to more informative trial results.

The FDA has been explicitly requiring well-designed patient-centric trials to assess the phase contribution of an agent to treatment outcomes in future perioperative regimens for resectable NSCLC. This paradigm-shifting event signals new scrutiny for determining the phase contribution of a single drug for perioperative cancer regimens, which aligns with the FDA’s recent focus on risk–benefit optimization in Project Optimus. Although it is unclear whether this principle of phase assessment will be extended to other cancer types, we advocate enforcing the standard to be responsive to patients’ needs for safe, effective, and cost-effective treatment options.

## Supplementary Information

**Additional file 1.** Why was the phase contribution not evaluated at the beginning?

## Data Availability

Not applicable.

## Abbreviations

NSCLC: Non-small cell lung cancer
FDA: The food and drug administration
HR: Hazard ratio
ICI: Immune checkpoint inhibitor
SOC: Standard of care
