# Supplementary material for: Toward phase contribution assessment in perioperative oncology: insights from NSCLC and a proposal for broader implementation
Source: Mil Med Res. 2025 Jul 31;12:43. doi: 10.1186/s40779-025-00622-2 (PMC12312453; doi:10.1186/s40779-025-00622-2)
Supplement: Supplementary file 1 — Additional file 1. Why was the phase contribution not evaluated at the beginning? [file 40779_2025_622_MOESM1_ESM.pdf]

Why was the phase contribution not evaluated at the beginning?

Owing to the complex pathogenesis and high likelihood of tumor recurrence, multidrug combinations and multistage treatment are very common in both clinical trials and practice. While intensified treatment is expected to demonstrate better efficacy, it may also increase safety concerns and treatment burdens. To evaluate the individual contributions of multiple drugs, a broad global regulatory consensus and guidelines on factorial analysis have been established. However, the need for within-trial assessment of the contributions of a single drug at different treatment stages has never been formally addressed. Consequently, the default approach is a 2-arm trial design to evaluate the accumulated risk-benefit profile of a perioperative regimen, leaving the independent contributions of neoadjuvant and adjuvant phases inadequately characterized.

The lack of such requirements stems from the evolution of cancer therapies. Previously, perioperative therapies were primarily limited to chemotherapy, typically 4 – 6 cycles, often supported by the evidence for adjuvant use. In the chemotherapy era, the phase-contribution question may not be a major concern. In a phase III trial from Felip et al. [1], no statistically significant difference in disease-free survival was observed when adding preoperative or adjuvant chemotherapy to surgery. This changed with immunotherapy. For the first time, the phase III KEYNOTE-522 trial introduced a significantly prolonged perioperative regimen of nearly 1-year of immune checkpoint inhibitors (ICIs) with limited prior adjuvant therapy evidence [2]. Unlike the direct cytotoxic effects of chemotherapy, ICIs work through intricate interactions with the immune system. These interactions may differ between the neoadjuvant and adjuvant stages, which are not yet fully understood. Emerging evidence suggests ICIs may have a greater impact in the neoadjuvant setting when the tumor and the lymph nodes are still in place [3]. The extended treatment duration and complex mechanism highlighted the need to reassess the benefit-risk profile at each stage of therapy in the immunotherapy era.

Additionally, implementing phase-specific assessments may pose practical challenges for pharmaceutical companies in terms of the research and development for perioperative tumor treatment. The application of component contribution assessment standards ideally requires that the performance of the perioperative regimen be compared with that of the neoadjuvant and adjuvant regimens via a 4-arm design. This approach will significantly increase costs, especially in perioperative settings that are generally characterized by long-term follow-up. Consequently, the large investment of time and finances may hinder the development of perioperative therapy and ultimately harm the interests of patients.

In 2018, at the end of phase 2 meeting, the FDA raised and highlighted the scientific need for phase assessment for the AEGEAN trial. However, before such requirements can be implemented officially, regulators must comprehensively assess the feasibility and necessity, as well as strike a balance between scientific needs and all other perspectives. Thus, the FDA never mandated phase assessment of a single cancer drug before the ODAC meeting for the AEGEAN trial.

References

1. Felip E, Rosell R, Maestre JA, Rodríguez-Paniagua JM, Morán T, Astudillo J, et al. Preoperative chemotherapy plus surgery versus surgery plus adjuvant chemotherapy versus surgery alone in early-stage non-small-cell lung cancer. J Clin Oncol. 2010;28(19):3138-45.
2. Schmid P, Cortes J, Pusztai L, McArthur H, Kümmel S, Bergh J, et al. Pembrolizumab for early triple-negative breast cancer. N Engl J Med. 2020;382(9):810-21.
3. Sinicrope FA, Turk MJ. Immune checkpoint blockade: timing is everything. J Immunother Cancer. 2024;12(8):e009722.
